# Myocarditis Associated With Herpes Zoster in a 91-Year-Old Woman: A Rare Cardiac Complication With Favorable Outcomes

**DOI:** 10.7759/cureus.96437

**Published:** 2025-11-09

**Authors:** Ana S Montenegro, José M Rodríguez Mirón, Roberto R Chapas

**Affiliations:** 1 Internal Medicine, Universidad Francisco Marroquin, Guatemala, GTM; 2 College of Medicine, Francisco Marroquín University, Guatemala, GTM; 3 Cardiology, Hospital Herrera Llerandi, Guatemala, GTM

**Keywords:** heart failure, herpes zoster, myocarditis, transthoracic echocardiogram, vaccine

## Abstract

Herpes zoster-associated myocarditis is an exceedingly rare and underreported complication of varicella-zoster virus reactivation. We describe a 91-year-old woman who developed a typical herpes zoster rash and, six days later, was admitted with clinical, laboratory, and echocardiographic evidence of acute heart failure suggestive of myocarditis. The patient improved rapidly with optimized heart failure therapy and escalation of valacyclovir, was weaned off oxygen and diuretics, and discharged in stable condition. A follow-up echocardiogram demonstrated marked recovery of left ventricular function. This case highlights the importance of recognizing rare cardiac complications of herpes zoster, such as myocarditis, and underscores the need for preventive strategies, including vaccination. Clinicians should maintain a high index of suspicion for myocarditis in patients presenting with new cardiac symptoms following herpes zoster infection.

## Introduction

Varicella-zoster virus (VZV), a member of the herpesviridae family, causes primary varicella (chickenpox) and, upon reactivation, herpes zoster. Reactivation occurs most often in older adults and immunocompromised individuals, with risk factors including advanced age, prolonged corticosteroid use, malignancy, solid organ transplantation, and stress [1]. Nevertheless, cases in immunocompetent patients have also been documented [2].

Herpes zoster typically presents with a prodrome of tingling, burning, or pain, followed by erythematous maculopapular lesions that evolve into vesicles and crust over in a dermatomal distribution, most frequently in the thoracic region [3]. Complications include herpes zoster ophthalmicus, meningoencephalitis, and postherpetic neuralgia (PHN), which occurs in approximately 13-25% of patients and is associated with a marked reduction in quality of life [4].

Beyond neurological complications, herpes zoster has also been associated with cardiovascular disease. Proposed mechanisms include direct viral invasion of the myocardium and vasculature, as well as immune-mediated inflammation, resulting in a prothrombotic state [5]. A study published in 2023 by Horev et al. found a 19% higher risk of all major adverse cardiac and cerebrovascular events (MACCE) within the first year following herpes zoster [6]. Reported cardiovascular complications include myocardial infarction, arrhythmias, and heart failure, with myocarditis documented only in sporadic case reports and its true prevalence remaining unknown [7].

We present a case of a 91-year-old woman who developed acute heart failure due to myocarditis shortly after herpes zoster. This report highlights the diagnostic approach, therapeutic considerations, and review of the available literature.

## Case presentation

A 91-year-old woman with a history of hypertension presented to the emergency department with an acute onset of retrosternal chest pain and dyspnea. Six days earlier, she developed a rash on the right side of her back and self-medicated with dexamethasone and diclofenac without improvement. The following day, as the rash extended to the right intercostal region, she was diagnosed with herpes zoster and prescribed valacyclovir 1 g every 12 h for five days, in addition to calamine lotion and topical lidocaine. Pain persisted, requiring paracetamol for partial relief. On the night of presentation, she awoke with severe oppressive chest pain (7/10, exacerbated by inspiration). Emergency services recorded an oxygen saturation of 87% and administered supplemental oxygen before transferring her to the hospital.

**Figure 1:**
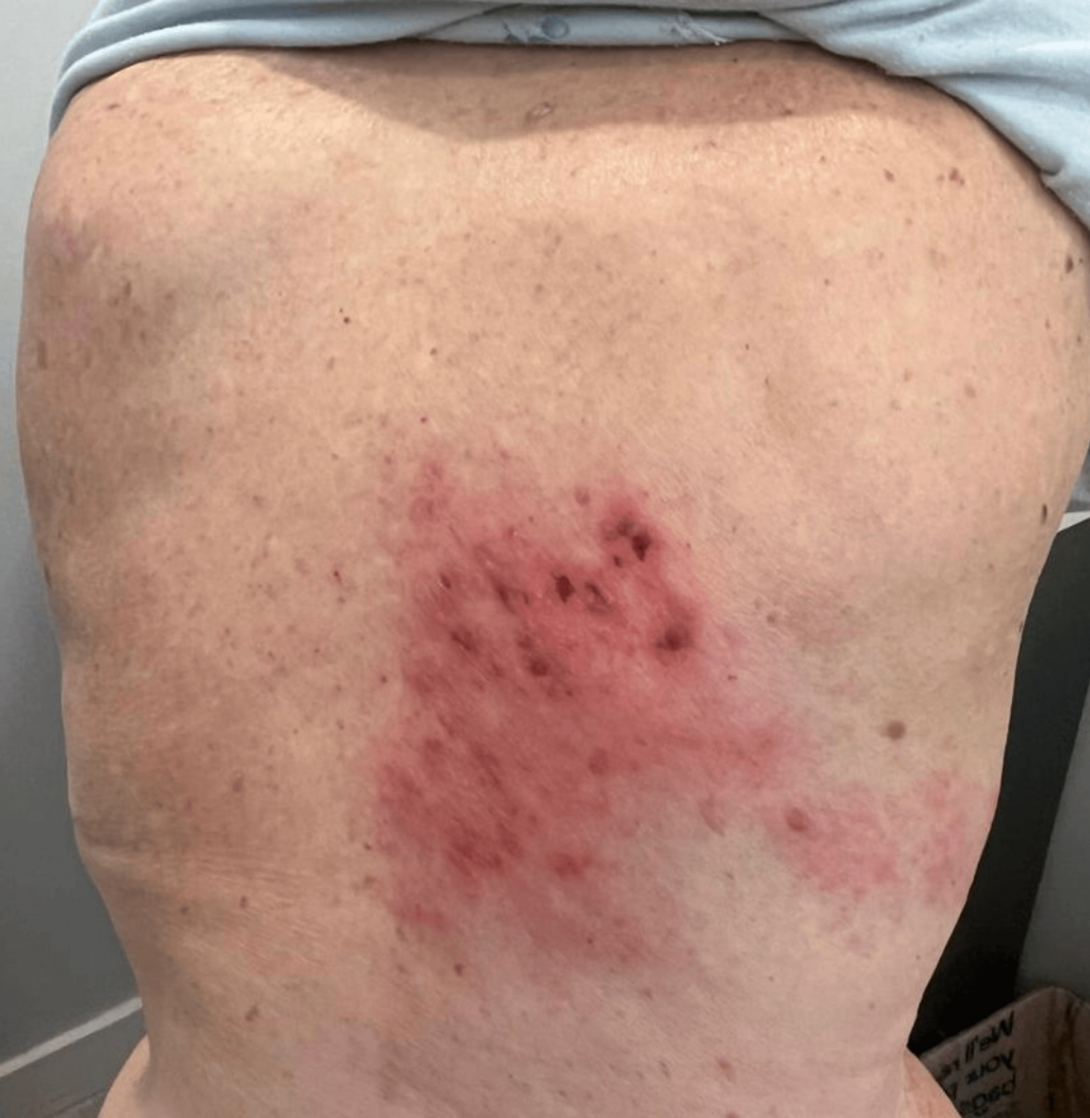
Herpes zoster rash on the patient’s back. Maculopapular and vesicular lesions on an erythematous base distributed along the right T6 dermatome, consistent with herpes zoster reactivation.

Upon arrival, she was afebrile (36.7°C), with a heart rate of 75 beats per minute, blood pressure of 125/70 mmHg, a respiratory rate of 22 breaths per minute, and an oxygen saturation of 94% on 2 L/min via nasal cannula. Pulmonary auscultation revealed bilateral crackles and expiratory wheezes, while cardiac examination was unremarkable. Dermatologic evaluation showed maculopapular lesions on an erythematous base distributed along the right T6 dermatome (Figure 1).

**Figure 2:**
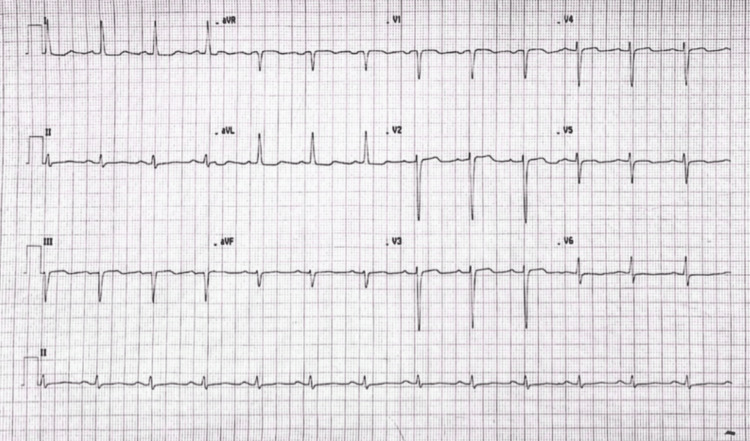
Electrocardiogram on admission A 12-lead ECG demonstrating sinus rhythm without acute ischemic changes.

Laboratory studies showed a normal white blood cell count, with mildly elevated C-reactive protein and erythrocyte sedimentation rate. D-dimer level was 695 ng/mL. Cardiac biomarkers were largely within normal limits, although N-terminal pro-B-type natriuretic peptide (NT-proBNP) was markedly elevated (11,828 pg/mL). A respiratory viral panel was negative. Serology was positive for both VZV IgM and IgG. Electrocardiography demonstrated sinus rhythm with no acute ischemic changes (Figure 2).

**Figure 3:**
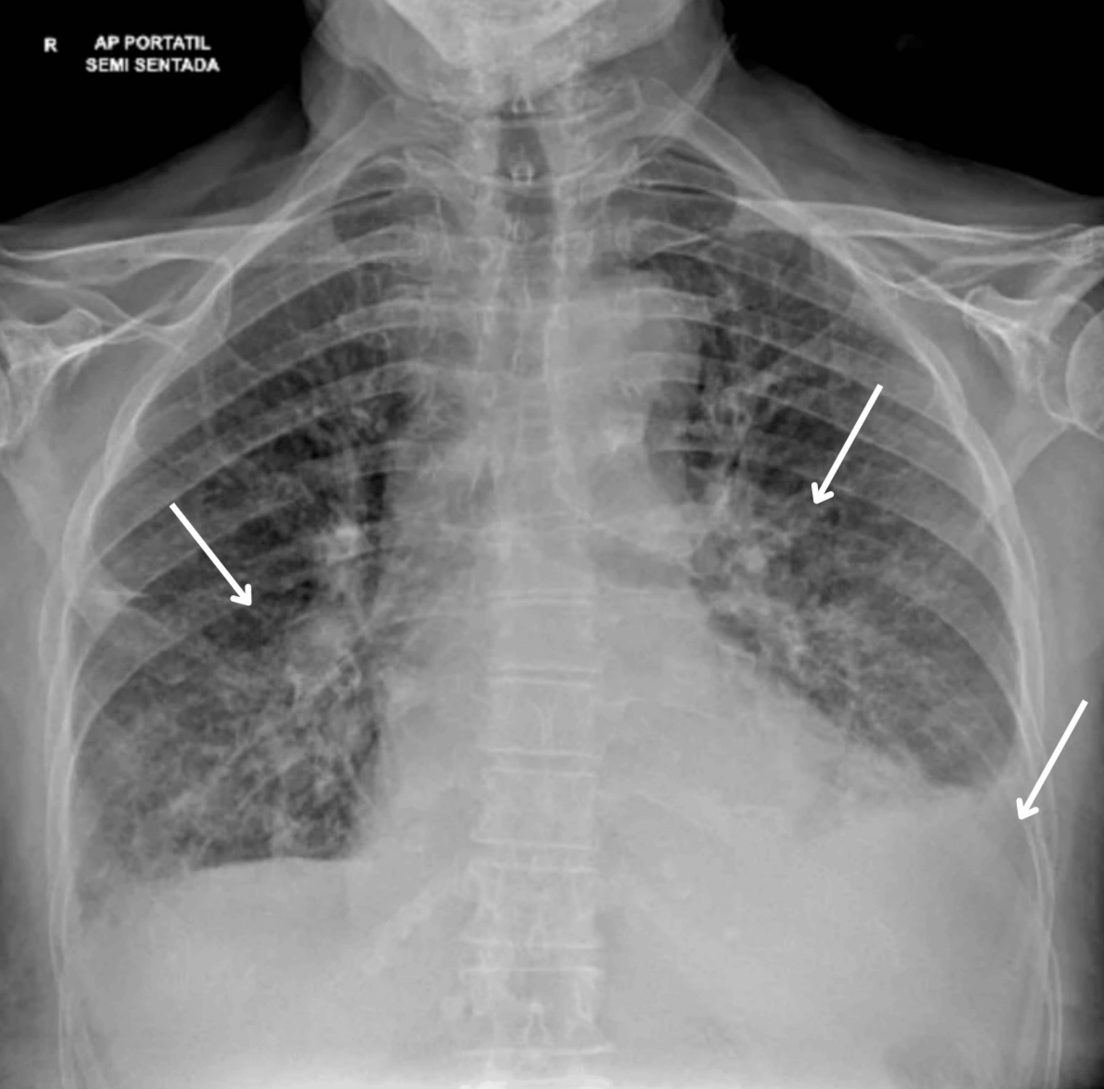
Chest radiograph on admission. The arrows highlight radiographic findings of pulmonary congestion and pleural effusion, which are significant because they demonstrate acute left-sided heart failure, in this case secondary to myocarditis. These findings support the clinical diagnosis and illustrate the degree of cardiac decompensation of the patient at presentation.

**Figure 4:**
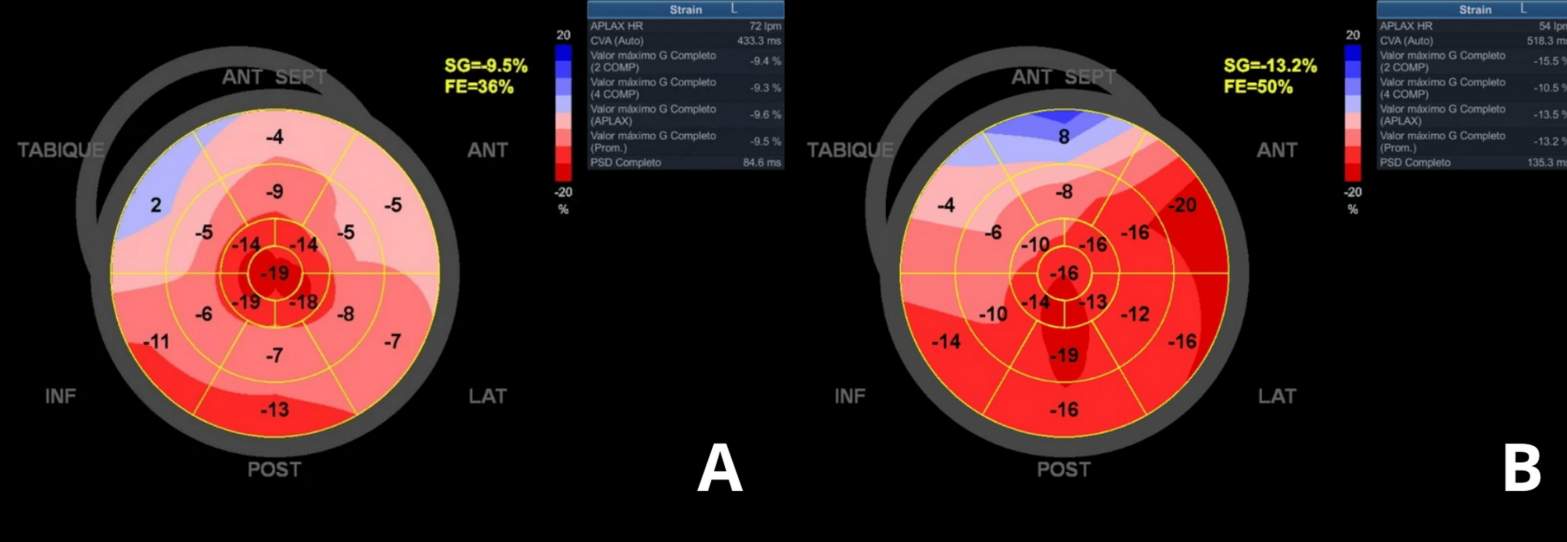
Strain analysis on transthoracic echocardiogram. (A) Admission: bull’s eye plot of global longitudinal strain showing markedly reduced values (-9.5%) and depressed left ventricular ejection fraction (LVEF 35%), consistent with impaired systolic function. (B) Two-week follow-up: improvement in global strain (-13.2%) with recovery of LVEF to 50%. TABIQUE: septal (refers to interventricular septum); INF: inferior LAT; LAT: lateral ANT; ANT: anterior SG; SG: strain global (GLS); GLS: global longitudinal strain; FE: fractional ejection (LVEF); LVEF: left ventricular ejection fraction

**Figure 5:**
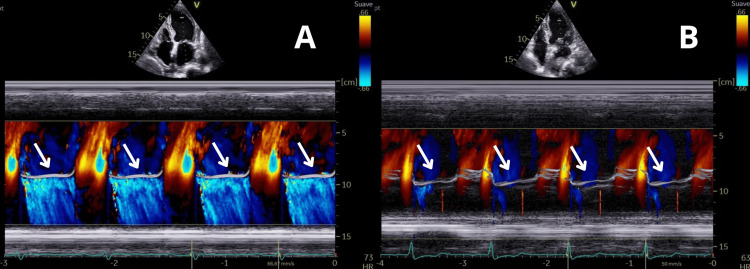
Mitral regurgitation on transthoracic echocardiogram. The arrows mark the mitral regurgitant jet on color Doppler imaging. This is significant as it illustrates functional mitral regurgitation associated with left-sided ventricular dysfunction, a common feature in acute myocarditis. However, since no prior echocardiographic study was available, it is uncertain whether the mitral regurgitation was present before and worsened or developed as part of the acute myocardial inflammation. The reduction in the regurgitant jet on follow-up emphasizes improvement in the cardiac function after treatment. (A) Admission: color Doppler imaging demonstrating severe mitral regurgitation with a prominent regurgitant jet. (B) Two-week follow-up: significant reduction in mitral regurgitation compared with baseline.

**Figure 6:**
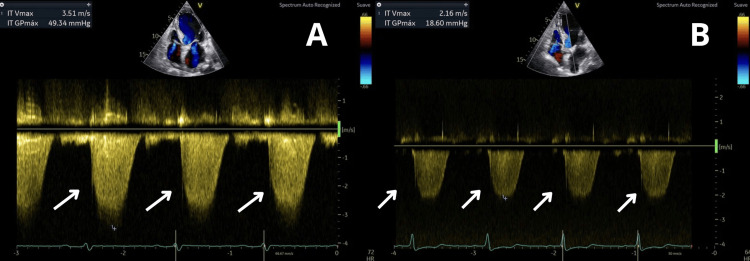
Tricuspid Doppler on transthoracic echocardiogram. The arrows identify the tricuspid regurgitant jet used to estimate pulmonary artery systolic pressure. The significance of demonstrating this is the presence and subsequent resolution of pulmonary hypertension, indicating the recovery of right ventricular function as myocarditis improves. (A) Admission: continuous-wave Doppler across the tricuspid valve showing elevated velocity (3.5 m/s) corresponding to a calculated right ventricular systolic pressure of ~49 mmHg, consistent with pulmonary hypertension. (B) Two-week follow-up: improvement with reduced velocity (2.1 m/s) and calculated gradient of 18.6 mmHg, indicating resolution of pulmonary hypertension. IT Vmax: tricuspid insufficiency maximum velocity; IT GPmax: tricuspid insufficiency maximum pressure gradient

Chest radiography showed pulmonary congestion with increased vascular markings and a left-sided pleural effusion (Figure 3). Transthoracic echocardiography demonstrated impaired systolic function with a left ventricular ejection fraction (LVEF) of 35% and severe valvular regurgitation. Strain analysis revealed global longitudinal strain of -9.5% (Figure 4, panel A). Severe mitral regurgitation was present (Figure 5, panel A), while tricuspid Doppler indicated pulmonary hypertension with a velocity of 3.5 m/s (gradient 49 mmHg) (Figure 6, panel A). This was the patient’s first documented echocardiographic evaluation; no baseline or prior echocardiogram was available for comparison.

Cardiac magnetic resonance (CMR) was performed and demonstrated myocardial edema and non-ischemic late gadolinium enhancement in the inferolateral wall, consistent with acute myocarditis. A coronary computed tomography (CT) angiography was performed, and ischemic heart disease was ruled out.

The working differential included acute coronary syndrome (ACS), pulmonary embolism, and viral pneumonia. ACS was less likely given normal troponin/creatinine kinase-MB and no ischemic electrocardiogram (ECG) changes. Pulmonary embolism was excluded by chest CT despite elevated D-dimer, and viral pneumonia was unlikely given the negative panel and absence of infiltrates. In the context of a recent herpes zoster rash and positive VZV serology, herpes zoster-associated myocarditis was considered the most likely etiology.

The patient was started on guideline-directed medical therapy for heart failure, including dapagliflozin, metoprolol, sacubitril/valsartan, spironolactone, and furosemide. The patient had initially been prescribed valacyclovir 1 g every 12 h as an outpatient, which is below the standard therapeutic dosing of 1 g every 8 h for herpes zoster. Following admission and recognition of myocarditis, the antiviral regimen was escalated to the correct oral dose of 1 g every 8 h, adjusted appropriately for her preserved renal function (glomerular filtration rate 75 mL/min/1.73 m^2^).

Over the following four days, the patient improved clinically, was weaned off oxygen, and NT-proBNP declined to 2,146 pg/mL by discharge. An echocardiogram performed prior to discharge already showed improvement in LVEF to 50%. At the two-week follow-up, echocardiography confirmed sustained recovery of LVEF (50%) with improved global strain (-13.2%) (Figure 4, panel B). Mitral regurgitation had decreased (Figure 5, panel B), and tricuspid valve velocity reduced to 2.1 m/s (gradient 18.6 mmHg), consistent with improvement in pulmonary hypertension (Figure 6, panel B). Residual moderate aortic regurgitation was noted. She was discharged in stable condition on optimized heart failure therapy and pregabalin for residual neuropathic pain.

## Discussion

VZV-associated myocarditis remains exceptionally rare. In a 2007 report, Kelly et al. described a patient who developed myocarditis five days after the appearance of a herpes zoster rash [8]. Another notable instance is a 2016 case by Elikowski et al., in which a 23-year-old male developed myopericarditis approximately one week after the onset of shingles [9]. More recently, in 2022, Park et al. reported fulminant hepatitis and myocarditis in a 39-year-old kidney transplant recipient with herpes zoster [10]. By contrast, most reported cases of VZV-related myocarditis occur in the setting of primary varicella (chickenpox), and several of those have had fatal outcomes, underscoring the greater severity of myocarditis during primary infection. Our case adds to the limited literature documenting myocarditis following VZV reactivation and is noteworthy for the favorable recovery achieved in an elderly patient.

To our knowledge, studies assessing its prevalence are lacking, highlighting the need for further investigation. A retrospective cohort study published in 2023 evaluating viral myocarditis found that although herpes zoster was among the least frequent viral causes, it carried the second-highest mortality rate [7].

There are currently no specific guidelines for diagnosing herpes zoster-associated myocarditis. Diagnostic evaluation follows the general myocarditis criteria outlined by the 2025 European Society of Cardiology (ESC), which combines clinical presentation, biomarkers, ECG abnormalities, and imaging findings. Echocardiography is usually the initial imaging tool, offering information on cardiac structure and function. Cardiac magnetic resonance imaging offers superior tissue characterization, allowing detection of myocardial and pericardial inflammation and patterns of fibrosis. Computed tomography is helpful in ruling out coronary artery disease. Endomyocardial biopsy is reserved for selected high-risk patients or when histological confirmation and viral identification are needed to guide therapy [11]. In the setting of recent herpes zoster reactivation, the temporal association and exclusion of other etiologies support the attribution to varicella zoster virus.

Antiviral therapy has been shown to reduce the duration of herpes zoster skin lesions and the severity of pain when initiated promptly, ideally within 72 h of rash onset. First-line options include acyclovir, valacyclovir, and famciclovir. While specific guidelines for herpes zoster-associated myocarditis are lacking, in complicated cases, intravenous acyclovir at 8-10 mg/kg every 8 h for 7-10 days is recommended, followed by oral therapy once clinical improvement is achieved [12].

In our patient, the initial outpatient prescription was valacyclovir 1 g every 12 h, which is below the standard therapeutic dosing of 1 g every 8 h for herpes zoster. Subtherapeutic dosing may have contributed to inadequate viral suppression and increased risk of complications. After admission, the regimen was escalated to the correct oral therapeutic dose of 1 g every 8 h, adjusted appropriately for her preserved renal function. Despite intravenous acyclovir being the typical choice in complicated presentations, our patient demonstrated rapid clinical improvement on appropriately dosed oral valacyclovir. This suggests that while intravenous therapy remains the preferred regimen, oral valacyclovir at full therapeutic dosing may be an effective alternative in carefully selected patients who are hemodynamically stable and able to tolerate oral medication.

Vaccination provides an effective strategy to reduce the incidence of herpes zoster and its complications. Two vaccines are currently available: the recombinant subunit vaccine, administered as two doses separated by two to six months, and the live attenuated vaccine, given as a single dose [13]. Vaccination is recommended in adults aged 50 years and older. Without immunization, approximately one-third of people are at risk of developing herpes zoster [13]. A South Korean cohort study, using data from 2012 to 2021, demonstrated that live zoster vaccination was associated with a lower risk of cardiovascular events (hazard ratio, 0.77), suggesting that vaccination may represent a valuable public health measure to reduce the cardiovascular disease burden [14].

## Conclusions

Herpes zoster-associated myocarditis is a rare but clinically significant complication of varicella-zoster virus reactivation. Clinicians should remain alert to the possibility of myocarditis in patients with new-onset cardiac symptoms following herpes zoster. Early recognition and supportive management can result in rapid clinical improvement and recovery of cardiac function. This case further highlights the importance of preventive strategies, such as herpes zoster vaccination, in reducing morbidity in high-risk populations.

## References

[1] Yamaoka-Tojo M, Tojo T (2024). Herpes zoster and cardiovascular disease: exploring associations and preventive measures through vaccination. Vaccines (Basel).

[2] Tseng HF, Bruxvoort K, Ackerson B (2020). The epidemiology of herpes zoster in immunocompetent, unvaccinated adults ≥50 years old: incidence, complications, hospitalization, mortality, and recurrence. J Infect Dis.

[3] Patil A, Goldust M, Wollina U (2022). Herpes zoster: a review of clinical manifestations and management. Viruses.

[4] Yawn BP, Saddier P, Wollan PC, St Sauver JL, Kurland MJ, Sy LS (2007). A population-based study of the incidence and complication rates of herpes zoster before zoster vaccine introduction. Mayo Clin Proc.

[5] Horev A, Horev A, Gordon-Irshai A, Gordon M, Andre N, Ifergane G (2023). Herpes zoster and long-term vascular risk: a retrospective cohort study. Sci Rep.

[6] Parameswaran GI, Drye AF, Wattengel BA, Carter MT, Doyle KM, Mergenhagen KA (2023). Increased myocardial infarction risk following herpes zoster infection. Open Forum Infect Dis.

[7] Kwok CS, Will M, Moertl D, Qureshi AI, Borovac JA (2023). The influence of diagnoses of specific viral infections on in-hospital mortality, length of stay and cost in patients admitted to hospital with a diagnosis of myocarditis: an analysis of the national inpatient sample. Rev Cardiovasc Med.

[8] Kelly E, Cullen G, McGurk C (2008). When an MI is not an MI: a case of varicella zoster myocarditis. Cardiology.

[9] Elikowski W, Marszalek A, Malek-Elikowska M, Ganowicz-Kaatz T, Mozer-Lisewska I (2016). Myopericarditis in a 23-year-old male with herpes zoster. Pol Merkur Lekarski.

[10] Park J, Kang M, Ha J, Lee H (2022). Fulminant hepatitis and myocarditis associated with varicella zoster virus infection in a kidney transplant recipient: a case report. Korean J Transplant.

[11] Schulz-Menger J, Collini V, Gröschel J (2025). 2025 ESC Guidelines for the management of myocarditis and pericarditis: developed by the task force for the management of myocarditis and pericarditis of the European Society of Cardiology (ESC). Eur Heart J.

[12] Gross GE, Eisert L, Doerr HW (2020). S2k guidelines for the diagnosis and treatment of herpes zoster and postherpetic neuralgia. J Dtsch Dermatol Ges.

[13] Tricco AC, Zarin W, Cardoso R (2018). Efficacy, effectiveness, and safety of herpes zoster vaccines in adults aged 50 and older: systematic review and network meta-analysis. BMJ.

[14] Lee S, Lee K, Oh J (2025). Live zoster vaccination and cardiovascular outcomes: a nationwide, South Korean study. Eur Heart J.

